# KEYNOTE‐811: Potential to revise first‐line therapy for metastatic human epidermal growth factor receptor 2‐positive gastric cancer

**DOI:** 10.1002/mco2.545

**Published:** 2024-05-07

**Authors:** Yuwei Ding, Ying Yuan, Shanshan Weng

**Affiliations:** ^1^ Department of Medical Oncology (Key Laboratory of Cancer Prevention and Intervention, China National Ministry of Education, Key Laboratory of Molecular Biology in Medical Sciences) The Second Affiliated Hospital Zhejiang University School of Medicine Hangzhou China; ^2^ Zhejiang Provincial Clinical Research Center for CANCER Hangzhou China; ^3^ Cancer Center of Zhejiang University Hangzhou China

**Keywords:** chemotherapy, gastric cancer, immunotherapy

## Abstract

The first‐line therapy pattern transition of metastatic HER2‐positive gastric cancer is shifting. The KEYNOTE‐811 study demonstrated that the addition of immunotherapy to the standard treatment of HER2‐targeted therapy and chemotherapy showed good results in terms of PFS, especially in subgroup patients with PD‐L1 CPS≥1. In the future, the first‐line therapy pattern of metastatic HER2‐positive gastric cancer will be radically changed based on ongoing randomized controlled clinical trials.

## Graphical abstract

The first‐line therapy pattern transition of metastatic human epidermal growth factor receptor 2 (HER2)‐positive gastric cancer (GC) is shifting. The KEYNOTE‐811 study demonstrated that the addition of immunotherapy to the standard treatment of HER2‐targeted therapy and chemotherapy showed good results in terms of progression‐free survival, especially in subgroup patients with programmed cell death ligand 1 combined positive score ≥ 1. In the future, the first‐line therapy pattern of metastatic HER2‐positive GC will be radically changed based on ongoing randomized controlled clinical trials.

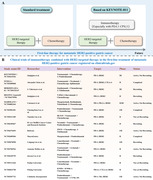

1

The KEYNOTE‐811 study, a prospective, randomized, controlled phase III trial, was recently published in *the Lancet*.^1^ It demonstrated that immunotherapy, along with human epidermal growth factor receptor 2 (HER2)‐targeted therapy and chemotherapy, was highly beneficial in treating HER2‐positive gastric cancer (GC).

Note that, 12%–20% of individuals diagnosed with GC exhibit HER2 amplification or overexpression, referred to as HER2‐positive. The primary techniques for HER2 expression detection are immunohistochemistry and, if required, fluorescence in situ hybridization (FISH). The patients enrolled in this study were identified as having locally advanced or metastatic HER2‐positive GC. The specific eligibility requirements included age over 18, without any previous treatment, and an Eastern Cooperative Oncology Group performance status score of 0 or 1. In a 1:1 random assignment, 698 patients were given either pembrolizumab or placebo, and both were to be taken every 3 weeks along with trastuzumab and traditional chemotherapy. Overall survival (OS) and progression‐free survival (PFS) were the 2 main results presented. The pembrolizumab cohort considerably outperformed the placebo cohort with a median PFS of 10.0 months versus 8.1 months (hazards ratio [HR] = 0.73; 95% confidence interval [95%CI] 0.60–0.87; *p* = 0.0002). The longer PFS by 3.6 months and a 30% lower risk of progressive disease was noticeable in the subgroup of patients with programmed cell death ligand 1 (PD‐L1) combined positive score (CPS) ≥ 1 (median PFS, 10.9 months versus 7.3 months; HR = 0.71; 95%CI 0.59–0.86). OS exhibited a more favorable trend in the pembrolizumab cohort than the placebo cohort (20.0 months vs. 16.8 months; HR = 0.84 [95%CI, 0.70–1.01]), as did in the PD‐L1 CPS ≥ 1 population (20.0 months versus 15.7 months; HR = 0.81 [95%CI, 0.67–0.98]). OS carried over to the final analysis since the pre‐established significance criteria were not satisfied. Both in the general population as well as in those who test positive for PD‐L1, the group treated with pembrolizumab exhibited a superior objective response rate (ORR), an extended median duration of response (DOR), and a greater percentage of responses lasting over 2 years. The ORR of the pembrolizumab cohort and the placebo cohort were 72.6% and 60.1%, respectively, and in the subgroup of PD‐L1 CPS ≥ 1 patients, the ORR was 73.2% versus 58.4%. The median DOR was 11.3 and 9.5 months, respectively. About 31% and 19% of respondents carried a response duration of ≥24 months. Regarding safety, the morbidity of ≥grade 3 therapy‐related adverse events (AEs) was 59% versus 51%. Grade 5 treatment‐related AE was seen in four (1.1%) versus three (0.9%) of the participants. Immunotherapy did not cause a discernible rise in immune‐related AEs.

Advanced GC patients who are positive for HER2 and have PD‐L1 CPS≥1 (Figure 1A) will be advised to undergo this combination treatment as their initial course of treatment, with additional data updates anticipated.

**FIGURE 1:**
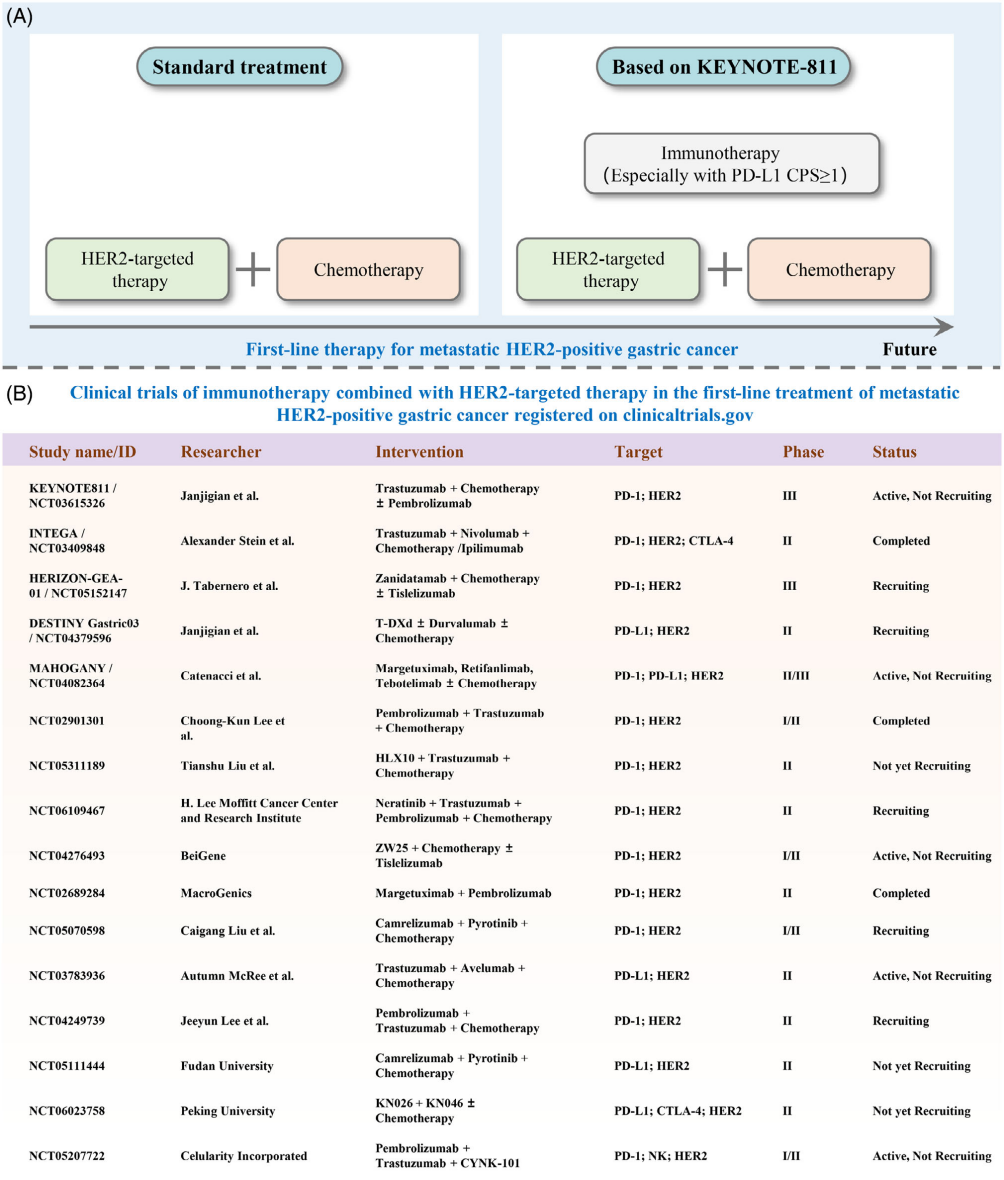
The shift in first‐line therapy for metastatic human epidermal growth factor receptor 2 (HER2)‐positive gastric cancer and the list of related ongoing clinical trials. (A) The current study demonstrated that adding immunotherapy to the conventional treatment of HER2‐targeted therapy and chemotherapy showed good results in terms of PFS, especially in subgroup patients with PD‐L1 CPS≥1. PD‐L1: programmed cell death protein; CPS: combined positive score; Chemo: chemotherapy. (B) The ongoing randomized controlled clinical trials of immunotherapy combined with HER2‐targeted therapy in the first‐line treatment of metastatic HER2‐positive gastric cancer. T‐DXd: trastuzumab‐deruxtecan; PD‐1: Programmed cell death‐1; PD‐L1: programmed death ligand 1; CTLA‐4: cytotoxic T‐lymphocyte‐associated antigen 4.

The Trastuzumab for Gastric Cancer (ToGA) study,^2^ conducted over a decade ago, marked a significant advancement in the field of targeted therapy for GC by showing that trastuzumab in conjunction with chemotherapy was effective. This groundbreaking research showed that this treatment combination could extend the OS by over 1 year and increase the ORR from 34.5% to 47.3% for patients with metastatic HER2‐positive GC. Even Nevertheless, following the ToGA study, there has been no further advancement in anti‐HER2 therapy for GC. The introduction of immunotherapy alters the landscape of advanced GC treatment, particularly for the HER2‐positive group. Furthermore, the application of antibody‐drug conjugate (ADC) medications is prevalent in the third‐line treatment. Trastuzumab deruxtecan (T‐dxd) has been licensed for second‐line and above therapy of HER2‐positive GC patients, based on the encouraging outcomes of the Destiny gastric 01.^3^ In the future, it may be tried to combine ADC medications with immunotherapy and/or tyrosine kinase inhibitors for HER2‐positive GC to attain greater efficacy and longer OS.

We are wondering if patients who are locally advanced and resectable could benefit from such a regimen in terms of improved tumor regression and pathologic complete response (pCR) rate. The KEYNOTE‐585 study^4^ released at the Congress of the European Society for Medical Oncology in 2023 gave a preliminary answer, which revealed that adding an inhibitor of programmed death‐ligand 1 (PD‐1) to the standard chemotherapy regimen significantly increased the pCR rate (12.9% vs. 2.0%, *p* < 0.00001). Unfortunately, when compared to placebo plus chemotherapy, no statistically significant improvement in event‐free survival was observed. A phase II clinical study^5^ reported at the American Society of Clinical Oncology found that camrelizumab + trastuzumab + CAPOX also showed good safety and tolerability during HER2‐positive GC's perioperative management, with 31.3% pCR rate, 100% R0 resection rate, and 77.3% ORR. Research such as the INNOVATION study and TRAP‐2 study is investigating the combination of chemotherapy and dual HER2‐targeted therapy. Concurrently, an exploratory perioperative trial on HER2‐positive GC has also been conducted using T‐dxd. The EPOC2003 Study, which evaluated the anti‐cancer effect of T‐dxd as neoadjuvant therapy in HER2‐positive GC patients, is in progress. The results of these studies mentioned above are all worth looking forward to.

At present, the world is paying great attention to the screening of HER2 in patients with cancer. One of the crucial requirements for treating GC in clinical practice is HER2 identification, which will significantly affect the course of treatment and its outcome. It should be highlighted that GC is heterogeneous (spatial and temporal heterogeneity) and that all patients, particularly those who have hematologic metastases, should have their HER2 status determined. A single test may not be sufficient for mixed GC patients, a second test or even metastatic detection may be necessary. When developing a treatment strategy for late‐line therapy, the re‐puncture biopsy is crucial, particularly in initially HER2‐negative patients. Simultaneously, CPS monitoring should not be disregarded. Circulating tumor DNA can be used as a tool for active monitoring of HER2 overexpression or amplification due to its relatively acceptable pathological consistency and non‐invasive benefits.

Researchers have long been interested in finding strategies to make therapy regimens for metastatic HER2‐positive GC more accurate. We have to commend the KEYNOTE‐811 study for its groundbreaking and historic findings, which offer excellent clinical supporting evidence for the strategies of immunotherapy combined with HER2‐targeted therapy, even though there are still some unanswered questions. These include the lack of disclosure sharing on the connection between PD‐L1 expression and efficacy, stratified data on patient populations of HER2 2+/FISH+ and HER2 3+, treatment options after PD (highly correlated with OS), and investigation of the connection between HER2 and the immune microenvironment.

In conclusion, the management of advanced HER2‐positive GC has changed along with the ToGA study, ADC drugs, the KEYNOTE‐811 study as well as the exploration of similar regimens in the perioperative period. We anticipate that current randomized controlled clinical trials (Figure 1B) will likely provide additional therapeutic choices by continuing to shed light. In the future, through the development of new drugs (chimeric antigen receptor T‐cell immunotherapy and vaccines), the combination of updated drugs is bound to further enhance treatment's sensitivity, safety, and efficacy.

## AUTHOR CONTRIBUTIONS

Yuwei Ding conceived and drafted the manuscript. Shanshan Weng and Ying Yuan are co‐corresponding authors and provided valuable discussion. All authors have read and approved the final manuscript.

## CONFLICT OF INTEREST STATEMENT

The authors declare no conflict of interest.

## FUNDING INFORMATION

This study was supported by the Fundamental Research Funds for the Central Universities (226‐2023‐00088), the Provincial Key R&D Program of Zhejiang Province (2021C03125), and the National Natural Science Foundation of China (82373415).

## ETHICS STATEMENT

Not applicable.

## Data Availability

Not applicable.
